# *In vitro* B cell experiments explore the role of CD24, CD38, and energy metabolism in ME/CFS

**DOI:** 10.3389/fimmu.2023.1178882

**Published:** 2024-01-08

**Authors:** Christopher W. Armstrong, Fane F. K. Mensah, Maria J. Leandro, Venkat Reddy, Paul R. Gooley, Saul Berkovitz, Geraldine Cambridge

**Affiliations:** ^1^Department of Biochemistry and Pharmacology, Bio21 Molecular Science and Biotechnology Institute, University of Melbourne, Melbourne, VIC, Australia; ^2^Department of Medicine, University College London, London, United Kingdom; ^3^Chronic Fatigue Service, Royal London Hospital of Integrated Medicine, University College Hospitals National Health Service Trust, London, United Kingdom

**Keywords:** chronic fatigue syndrome, B cells, metabolomics, CD24, metabolism

## Abstract

**Introduction:**

Disturbances of energy metabolism contribute to the clinical manifestations of myalgic encephalomyelitis/chronic fatigue syndrome (ME/CFS). Previously, we found that B cells from ME/CFS patients have an increased expression of CD24, a modulator of many cellular functions including those of cell stress. The relative ability of B cells from ME/CFS patients and healthy controls (HC) to respond to rapid changes in energy demand was compared.

**Methods:**

CD24, the ectonucleotidases CD39 and CD73, the NAD-degrading enzyme CD38, and mitochondrial mass (MM) were measured following cross-linking of the B cell receptor and costimulation with either T-cell-dependent or Toll-like-receptor-9-dependent agonists. The levels of metabolites consumed/produced were measured using 1H-NMR spectroscopy and analyzed in relation to cell growth and immunophenotype.

**Results:**

Proliferating B cells from patients with ME/CFS showed a lower mitochondrial mass and a significantly increased usage of essential amino acids compared with those from HC, with a significantly delayed loss of CD24 and an increased expression of CD38 following stimulation.

**Discussion:**

The immunophenotype results suggested the triggering of a stress response in ME/CFS B cells associated with the increased usage of additional substrates to maintain necessary ATP levels. Disturbances in energy metabolism in ME/CFS B cells were thus confirmed in a dynamic in vitro model, providing the basis for further mechanistic investigations.

## Introduction

Myalgic encephalomyelitis/chronic fatigue syndrome (ME/CFS) is a poorly understood chronic disease despite a prevalence of approximately 0.9% and an often debilitating course (1–3). Fatigue, post-exertional malaise, brain fog, and unrefreshing sleep are hallmark symptoms of the disease that are required to meet the diagnostic criteria. The reliance on symptom-based diagnosis is due to the absence of objective biomarkers, which has contributed to our gap in disease knowledge (4). Disruptions within the nervous, neuroendocrine, and immune systems, consequent to an infection or other environmental triggers, are generally thought to underlie ME/CFS, but precise mechanistic pathways resulting in the apparent dysregulation of multiple homeostatic pathways are not yet known (5–8).

The most consistent evidence for a biomedical basis for ME/CFS comes from studies of energy metabolism with metabolomic studies of the blood, urine, and feces of patients with ME/CFS describing clear alterations in lipid, urea, and glycolytic cycles (7, 9–13). The involvement of the immune system is also strongly suspected (14). The results of investigations of immune system parameters such as immunophenotype of T and B cells differ widely, but minor alterations in both T (MAIT cells) and B cell subpopulations have been described (15, 16). Some reports also suggest a pro-inflammatory profile in blood and CSF cytokines, which may be linked with metabolic activity (17, 18), whereas other investigations have proved negative (19). Overall, however, there is no clear or consistent body of evidence for specific abnormalities of immune functionality in patient cohorts as a whole, although it remains likely that immune system homeostasis is disturbed (14).

Previously, we reported that the expression of the glycoprotein CD24 (heat-stable antigen or nectadrin) was increased (both frequency and expression) on IgD+ naïve and memory B cell subsets in ME/CFS peripheral blood compared with age-matched healthy controls (20). Using B cells from healthy donors, we further demonstrated a novel association between CD24 positivity and energy metabolism, namely, phosphorylation of AMP-activated protein kinase (AMPK) in unswitched memory (IgD+IgM+CD27+) B cells.

The discovery of a possible link between the expression of CD24 on B cells with utilization of alternate pathways of ATP generation prompted us to investigate the B cell metabolic function in ME/CFS patients. Here we report differences in metabolic function of purified B cells from patients with ME/CFS and healthy controls. We used flow cytometry to analyze the CD24 expression as well as extracellular purinergic signaling (including the ectonucleotidases CD39 and CD73 and the NAD degrading enzyme CD38) throughout in response to cross-linking of the B cell receptor (BCR) and co-stimulation with either T-cell-dependent (TD) or Toll-like-receptor-9-dependent (TLR9D) ligands. The relative mitochondrial mass over consecutive cycles of proliferation was also calculated using flow cytometry. Levels of selected metabolites consumed/produced were measured using ^1^H nuclear magnetic resonance (NMR) spectroscopy and analyzed in relation to cell growth and immunophenotype.

## Materials and methods

### Patients and controls

Patients diagnosed with ME/CFS fulfilling the International (based on Canadian) Consensus Criteria (21) were selected for the study at an ME/CFS referral center, namely, the Royal London Hospital of Integrated Medicine, UCLH NHS Foundation Trust (under the care of Dr. S. Berkovitz). A total of eight ME/CFS patients (six female (F) and two male (M); median age, 33 years; range, 22–52 years) and seven healthy controls (HC) (five F and two M; median age, 33 years; range, 23–63) were included. Patients with a confirmed history of autoimmune disease or receiving immunosuppression were excluded, as well as those who had a history of depression (HADS >10/21). All were Caucasian, and the patients completed VAS scores at the time of blood taking (where 0 is no symptoms and 10 is most severe). The median scores were 7.3 for fatigue, 4.3 for cognitive impairment, and 4.0 for pain. This study has been approved by the NRES Committee London-City Road and Hampstead Research Ethics Committee (REC reference: 14/LO/0388).

### Purification and culture of B cells from peripheral blood samples

Peripheral blood mononuclear cells (PBMCs) were isolated from heparinized whole-blood samples by centrifugation over Lymphoprep (Stemcell™ Technologies, Vancouver BC). The B cells were enriched by negative selection using the EasySep™ Human B cell isolation kit (Stemcell™ Technologies, Vancouver, BC, Canada). The negatively isolated B cells were cultured in the presence of TD stimulus comprising anti-CD40 (LEAF™ Purified anti-human, Biolegend, San Diego, CA, USA), anti-IgM (AffiniPure F(ab’)2 Fragment Goat Anti-Human IgM, Fc5μ fragment specific, West Grove, PA, USA), and IL2 (Human, PeproTech EC Ltd., Rocky Hill, NJ, USA) or through TLR9D with CpG oligodeoxynucleotides (ODN 2006, InVivogen, San Diego, CA, USA) + anti-IgM and IL2. The unstimulated wells were cultured in complete medium alone.

### *In vitro* assay conditions and sampling following stimulation with T-cell-dependent and TLR9-dependent agonists

Purified B cells (2 × 10^5^/well in triplicate in 96-well culture plates) from HC (*N* = 7) and ME/CFS patients (*N* = 6) were cultured in 1.5-mL complete medium (RPMI-1640; Sigma-Aldrich, St. Louis, MO, USA, supplemented with 10% fetal bovine serum (FBS); Labtech International, Heathfield, UK) for up to 6 days in 24-well culture plates with transwell inserts (Corning, New York, NY, USA) plus TD or TLR9D stimulation in triplicate. Supernatants were collected on days 1, 3, and 6. Due to the low numbers of isolated B cells in some HC and ME/CFS samples, TLR9D stimulation was used for all individuals, with negative controls (in the absence of stimulation), and B cells stimulated by the TD pathway were included when the cell number allowed.

### Proliferation and staining of mitochondrial mass in B cell cultures from HC and ME/CFS patients

To confirm proliferation following stimulation, isolated B cells were stained with carboxyfluorescein succinimidyl ester (CFSE) (Biolegend, San Diego, CA, USA) and cultured for 6 days. Briefly, the cells were incubated with CFSE for 10 min at 37°C, quenched with cold complete medium, and washed for 5 min at 300 × *g*. CFSE-stained PBMCs/B cells were cultured in the presence of TD and TLR9D or with culture medium. Flow cytometry was then used to distinguish B cell subsets (CD19, CD27, and IgD) and cycles of proliferation.

To measure the mitochondrial mass, cultured B cells were incubated with 22 nM MitoTracker™ Red FM (ThermoFisher Scientific, Waltham, MA, USA) in preheated (37°C) RPMI medium 1640 without serum for 30 min at 37°C. The cells were then washed (5 min 300 × *g* at RT) with RPMI, resuspended in PBS, and stained for 20 min with anti-CD19-Alexa Fluor 700, anti-IgDBV421, and anti-CD27-APC. LIVE/DEAD^®^ Fixable Aqua Dead Cell Stain (ThermoFisher Scientific, Waltham, MA, USA) was used to gate out dead cells.

### Immunophenotype analysis

Flow cytometry was used to follow changes in classical B cell immunophenotype markers (CD19, IgD, IgM, and CD27) as well as those involved in energy pathways including CD24, CD39, CD73, and CD38 throughout *in vitro* culture. B cell numbers, B cell growth (total mass), and viability were also calculated using flow cytometry. Briefly, PBMCs or B cells were stained for 20 min with fluorescent conjugates of monoclonal antibodies to CD19-Alexa Fluor 700, IgD-BV421, and IgM-BV605 (BD Biosciences, San Jose, CA, USA), CD27-APC and CD24-APC eFluor780 (eBioscience, San Diego, CA, USA), CD39-FITC, CD73-PE, and CD38-PerCP.Cy5.5 (Biolegend, San Diego, CA, USA), and a viability marker (LIVE/DEAD™ Fixable aqua dead cell stain, ThermoFisher Scientific, Waltham, MA, USA). The cells were washed and resuspended in PBS and acquired within 24 h on a BD LSR FortessaTMX-20. Total mass was calculated by combining the mass of debris, dead cells, and live cells in each culture well using the following formula: (FSC × mean number viable + FSC × mean number dead + FSC × mean number debris). Compensation beads (BD, Biosciences, San Jose, CA, USA) were used to optimize the fluorescence compensation settings for multicolor flow cytometric analysis. A minimum of 100,000 events in the lymphocyte gate was collected.

### Determination of metabolites during *in vitro* culture using ^1^H nuclear magnetic resonance

In total, 500 µL of culture supernatants was sampled at days 1, 3, and 6, of which 200 µL was combined with 200 µL of ice-cold methanol-d_4_ (Sigma-Aldrich, St. Louis, MO, USA) and allowed to rest for 3 min; then, 200 μL ice-cold deuterated chloroform-d (Sigma-Aldrich, St. Louis, MO, USA) was added and mixed by vortexing. The samples were then centrifuged at 4°C to produce a biphasic mixture with a hydrophilic phase of water/deuterated methanol and a lipophilic phase of deuterated chloroform. A 300-μL sample of the top hydrophilic layer was added to 300 µL of 200 mM sodium phosphate in D_2_O (pH 7) containing 2 mM DSS and 0.2% (w/v) sodium azide. Then, 550 μL of supernatant was transferred to a 7-inch 5-mm 507-grade NMR tube and run on 700 MHz (Bruker Avance IIIHD 700) for NMR analysis. The metabolites in samples from cultures were analyzed as previously described (22). Fold changes in metabolites were calculated between days 1 and 3 and between days 3 and 6. Day 1 results were expressed as concentrations (in mM).

## Results

### Kinetics of CD24 on purified B cells after culture with TD and TLR9D agonists

**Figure 1:**
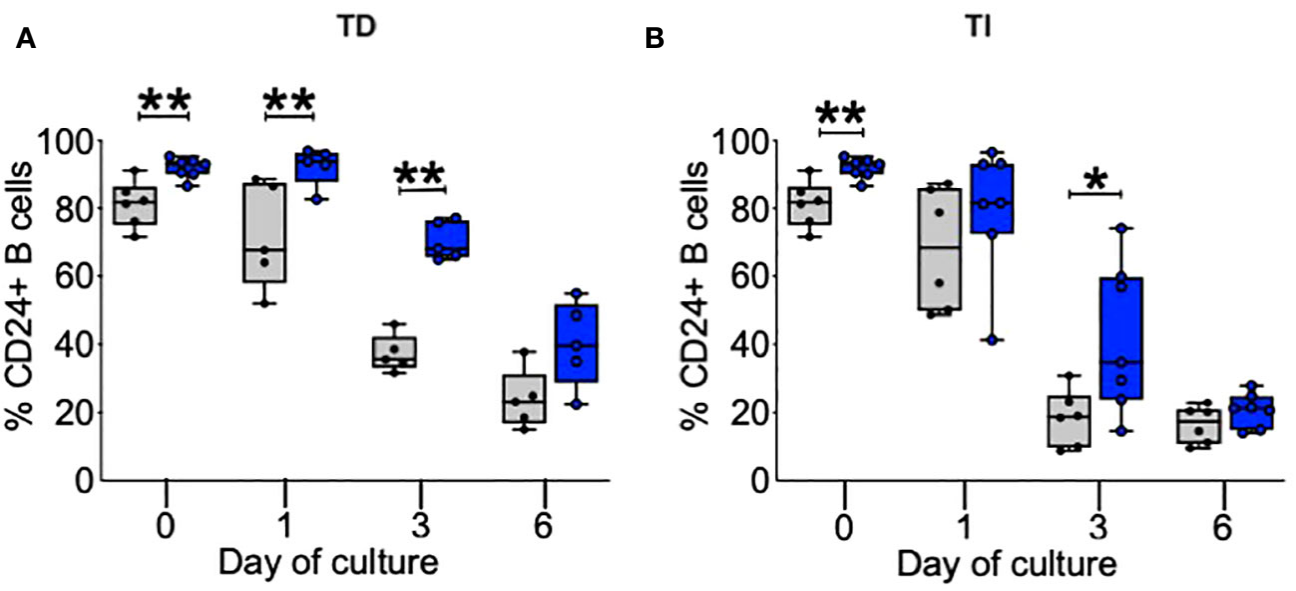
Percentage of B cells positive for CD24 after *in vitro* stimulation. %CD24+ B cells are shown at baseline, day 1, day 3, and day 6 after T-cell-dependent (TD) **(A)** or Toll-like-receptor-9-dependent (TI) ligands **(B)**. The box and whiskers show median, maximum, and minimum values for healthy controls (gray bars, *N* = 6) and myalgic encephalomyelitis/chronic fatigue syndrome patients (blue bars, *N* = 7); *p < 0.05, **p < 0.01 (Mann–Whitney *U*-test).

In our initial studies of B cells from patients with ME/CFS and HC, the frequency of CD24+ cells and CD24 expression was higher on peripheral blood B cells bearing IgD in the patient group (16). As shown in **Figure 1**, even in this small group pf patients, prior to culture the frequency of CD24 was significantly higher (*p* < 0.01) on B cells from ME/CFS patients compared to HC, confirming our previous studies of circulating B cells in peripheral blood. CD24 is rapidly lost after successful entrance into cycles of proliferation, and by day 3 of culture the percentage of CD24+ B cells from HC had reduced significantly in response to both stimuli. The higher percentage of CD24+ B cells in cultures from ME/CFS patients persisted, being evident in TD-stimulated cultures at days 1 and 3 (**Figure 1A**) and in TLR9D-stimulated wells (**Figure 1B**) at 3 days of culture. By day 6 of culture, the percentage of CD24+ B cells in cultures from ME/CFS patients was similar to that of HC (**Figure 1**). The mean levels of CD24 expression (MFI) decreased rapidly following stimulation but were similar between the patient and control groups (data not shown). The *in vitro* experiments support our previous findings of a higher CD24 frequency in peripheral blood B cells from ME/CFS patients. The lag in loss of CD24 from a high percentage of ME/CFS B cells may reflect their relatively slower rate of entry into the cell cycle compared with B cells from HC.

### Frequencies and expression of purinergic pathway markers on B cells from HC and ME/CFS patients

**Figure 2:**
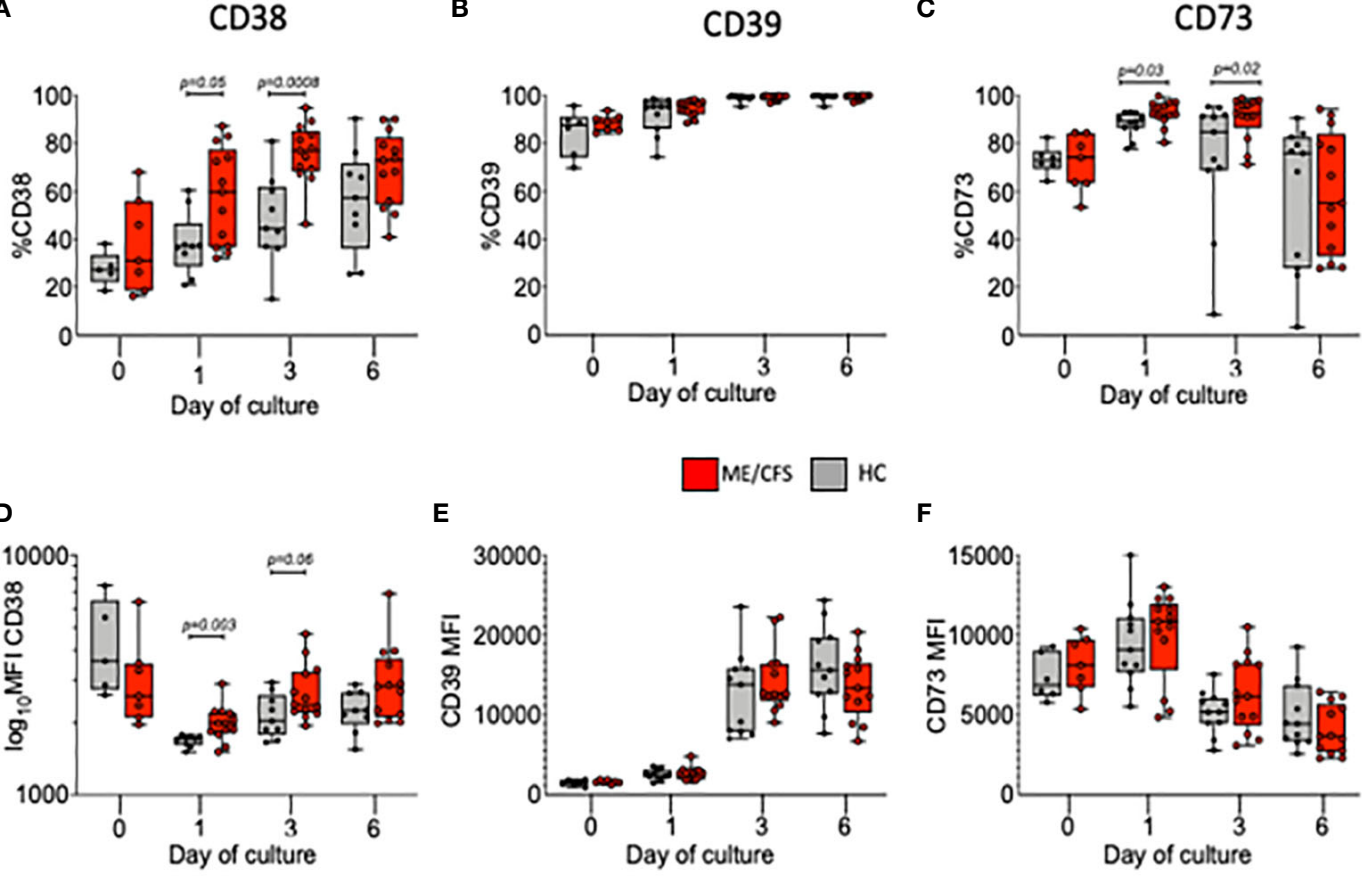
Percentage and MFI of live B cells positive for CD38, CD39, and CD73 following *in vitro* stimulation. The percentages of live B cells positive for CD38 **(A)**, CD39 **(B)**, CD73 **(C)**, and MFI **(D–F)** are shown at baseline (0) and days 1, 3, and 6 for healthy controls (HC; gray bars) and myalgic encephalomyelitis/chronic fatigue syndrome (ME/CFS; red bars). The results from both T-cell-dependent and Toll-like-receptor-9-dependent stimuli were combined. The box and whiskers show median, maximum, and minimum values for HC (*N* = 6) and ME/CFS patients (*N* = 7). The exact *p*-values, as shown, were calculated using Mann–Whitney *U*-test.

A series of ectoenzymes regulate the extracellular purinergic signaling pathways through their ability to modulate the levels of ATP and adenosine. Degradation to adenosine is associated with the activation of anti-inflammatory pathways. We therefore followed the frequencies and MFI (expression) of the ectonucleotidases CD39 and CD73 and of the NAD-degrading enzyme cyclic ADP ribose hydrolase (CD38) on live B cells cultured from HC and ME/CFS patients (**Figure 2**). The results from each stimulus were combined due to low cell numbers. At baseline, these three surface markers were found in similar frequencies in both HC and ME/CFS patients with no differences in expression. During culture, however, the frequencies (%) of both CD38+ and CD73+ B cells were significantly higher in ME/CFS compared with HC cultures (**Figures 2A, C**). The mean expression (MFI) of CD38, but not CD73, was found to be significantly increased in cultures from ME/CFS patients compared with HC at days 1 and 3, which corresponds with the period of most proliferation (**Figures 2D, F**). The expression (MFI) of CD39 was not different between patients and controls (**Figure 2E**).

### Mitochondrial mass of B cells from HC compared with ME/CFS following TD and TLR9D stimulation

**Figure 3:**
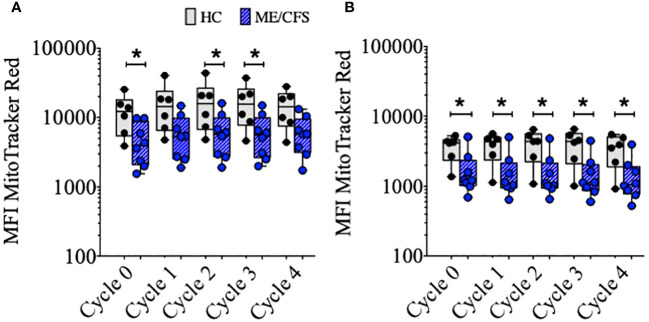
Mitochondrial mass (MFI of MitoTracker™ Red) of CD19+B cells over sequential cycles of proliferation. Purified B cells were stimulated with T-cell-dependent **(A)** or Toll-like-receptor-9-dependent agonists **(B)**. The results for healthy controls (HC) (gray bars, *N* = 6) and myalgic encephalomyelitis/chronic fatigue syndrome (ME/CFS) (blue bars, *N* = 8) are shown. The comparison between HC and ME/CFS patients’ results was performed using Mann–Whitney *U*-test; *p < 0.05.

Mitochondrial biogenesis (increase in volume or number of mitochondria) occurs as a response to stress and thus thought to be protective for the cell in the face of potential reactive oxygen species (ROS)-mediated damage. In murine B cells, mitochondrial mass (MM) has been shown to be associated with the fate of B cells following BCR crosslinking, with differentiation to class switch and entry to germinal centers associated with higher mitochondrial mass compared with T-independent second signaling which shows more proliferation and differentiation to (IgM+) plasma cells (23–25). In accord with these findings, the results described here show the greatest increase in MM associated with T-dependent signaling. TLR9D stimulation resulted in B cells attaining a relatively lower MM which was associated with more cycles of proliferation (**Supplementary Figure S1**). Therefore, to take into account the differences in B cell kinetics following stimulation with TD and TLR9D agonists, we measured MM in relation to each cycle of proliferation in the transwell B cell cultures over four proliferation cycles in B cells from six HC and eight ME/CFS patients (**Figure 3**). At baseline and over each cycle, MM was found to be significantly lower in ME/CFS compared to HC B cells, in most cycles following TD stimulation and all cultures following TLR9D stimulation. Differences in MitoTracker Red MFI between TD and TLR9D reflect higher MM following TD, as previously discussed.

### Comparison of glucose uptake and lactate production in B cell cultures: Relationship with immunophenotype

**Figure 4:**
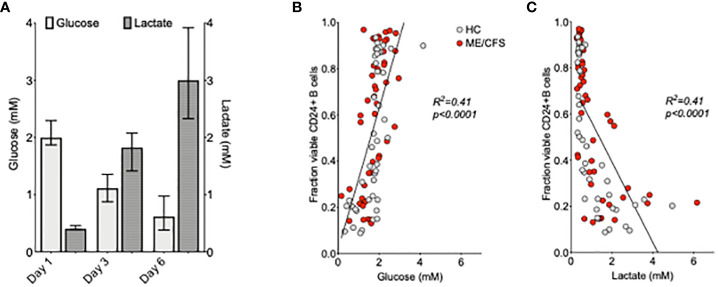
Relationship between glucose and lactate concentrations and %CD24+ B cells after the *in vitro* culture of purified B cells. Median ( ± IQR) of glucose and lactate concentrations (mM) measured at days 1, 3, and 6 in the supernatant of purified B cell cultures from six healthy controls following Toll-like-receptor-9-dependent (TLR9D) stimulation **(A)**. The proportion of CD24+B cells in live cell gates was plotted against the concentrations of glucose **(B)** and lactate **(C)** in the culture supernatants of 42 cultures from HC and 48 from myalgic encephalomyelitis/chronic fatigue syndrome patients. The results for cultures of B cells incubated with medium alone and T-cell-dependent and TLR9D stimulations were pooled. Statistical significance was calculated using linear regression and Pearson’s correlation coefficient as shown.

Glucose consumption and lactate production are important components in ATP generation through (anaerobic) glycolysis. Glucose and lactate concentrations (mM) were measured at days 1, 3, and 6 in the supernatant of purified B cell cultures from six HC using ^1^H NMR spectroscopy (**Figure 4A**). There was a clear decrease of glucose levels in conjunction with an increase of lactate concentrations between days 1 and 3 and days 3 and 6 of culture following TLR9D stimulation. The relationship between a direct measure of glucose and lactate throughout B cell maturation was also tested against changes in B cell phenotypic markers. CD markers previously measured at baseline (as shown in **Figure 2**) were analyzed in relation with glucose usage and lactate production. It was found that only fractions of viable CD24+ B cells, but none of the other CD markers tested, correlated strongly with glucose (**Figure 4B**) and lactate (**Figure 4C**) concentrations in both HC and ME/CFS patients irrespective of time of sampling (data not shown). There was no difference between the results for HC and ME/CFS patients.

### Culture conditions and measurement of cell growth

**Figure 5:**
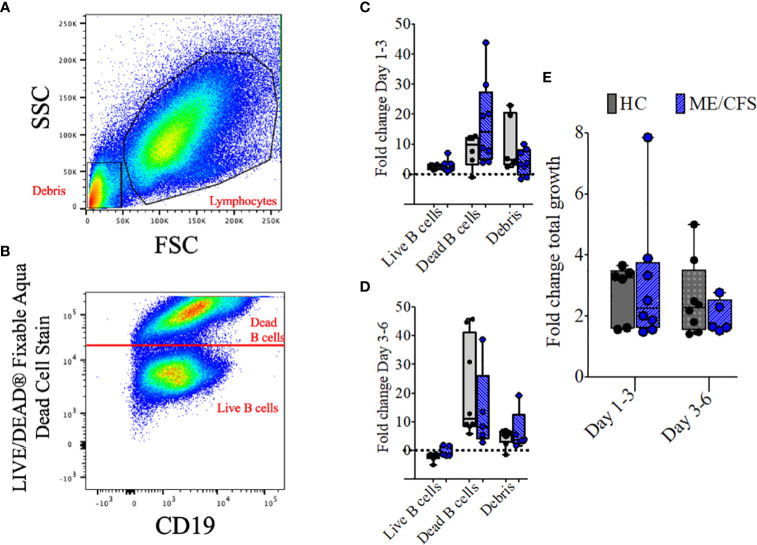
Representative plots and fold change in culture components and fold change in total growth. Representative dot plots show the gating strategy for lymphocytes and debris **(A)** and live and dead B cells **(B)** after 6 days of culture with T-cell-dependent and Toll-like-receptor-9-dependent stimulations (combined). Fold change differences in growth are shown for live B cells, dead B cells, and debris between days 1–3 **(C)** and days 3–6 **(D)**. The three populations were also combined and shown as fold change of total growth in mass between days 1–3 and days 3–6 **(E)**. The box and whiskers show median, maximum, and minimum values for a total of eight healthy controls and five myalgic encephalomyelitis/chronic fatigue syndrome patients.

To compare the metabolomic analysis of culture supernatants of patient and control cultures, it was necessary to take account of the proportions of live cells, dead cells, and debris within the culture milieux as differences may influence the results. The fold changes in all three parameters and total growth of HC and ME/CFS B cell cultures were compared over time. Representative plots of the gating strategy used in a day 6 B cell culture from a healthy control are shown in **Figure 5**. In **Figure 5A**, lymphocyte and debris gates are shown, and in **Figure 5B** dead cells were distinguished from live cells using LIVE/DEAD fixable stain. Growth, irrespective of TD or TI stimulation, was measured, and only positive cultures were included. **Figures 5C, D** show the fold change in mass for cultures from HC and ME/CFS patients for all three parameters between days 1–3 and days 3–6. The fold change in total mass was also calculated (**Figure 5E**). There was no significant difference between any of the parameters in HC vs. ME/CFS patients.

### Measurement of metabolites in culture supernatants of proliferating B cells from HC and patients with ME/CFS

Changes in metabolites (concentrations and fold changes over time) in cultured B cells from HC and ME/CFS patients were measured with respect to B cell growth. As shown in a representative experiment using B cells from a HC sample (**Supplementary Data S2**), B cells increased their cell size (forward scatter) at day 3 compared with day 1, while at 6 days the cell size decreased compared with that on day 3. Side scatter (internal granularity/complexity) was shown to be the highest at day 6, compared to days 1 and 3, reflecting differentiation to plasmablasts. Changes in CD markers and metabolites were therefore measured in relation to B cell growth in cultures during the critical growth phase of days 1 to 3 and then days 3 to 6. As both TD and TLR9D stimulation induced B cell growth, the results from supernatants were combined as the numbers were small.

In **Table 1**, the significant differences between ME/CFS and HC are summarized for metabolites present in the culture medium in relation to CD markers and cell mass. Of note is the significant elevation of CD38 on live ME/CFS B cells and the reduced fold change of CD38 markers on dying ME/CFS B cells. CD38 thus appears to be linked with B cell survival in ME/CFS patients. Finally, the proportion of live B cells in ME/CFS as measured by live cell mass appears to decrease less rapidly than in healthy controls.

**Table 1:** The significant differences (shown in *p*-values) between proliferating B cells from myalgic encephalomyelitis/chronic fatigue syndrome and healthy controls are summarized.

	Conc/intensity	%	FC	FC of %
Acetate	0.1	0.1	0.4	1.0
Alanine	0.8S	0.8	0.3	0.9
Arginine	0.2	0.1	0.2	0.9
Aspartate	0.7	0.8	0.4	0.9
Creatine	0.2	0.1	0.2	0.9
Creatinine	0.2	0.1	0.4	1.0
Formate	0.7	0.8	0.7	1.0
Glucose	0.8	0.9	0.4	0.9
Glutamate	0.4	0.4	0.1	1.0
Glycine	0.2	0.1	0.3	1.0
Histidine	0.3	0.4	0.4	1.0
Isoleucine	0.7	0.8	0.3	0.9
Lactate	0.7	0.7	0.5	0.9
Leucine	0.2	0.1	0.2	0.9
Lysine	0.1	0.1	0.4	1.0
Phenylalanine	0.2	0.3	0.2	1.0
Proline	0.8	0.8	0.9	1.0
Threonine	0.6	0.7	0.4	0.9
Tryptophan	0.4	0.5	0.1	0.9
Tyrosine	0.2	0.2	0.1	1.0
Valine	0.2	0.2	0.1	1.0
Hydroxyproline	0.2	0.2	0.6	1.0
Total metabolite	0.7		0.4	
CD24 on living cells	0.3	0.5	0.4	0.9
CD27 on living cells	0.5	0.4	0.2	0.7
CD38 on living cells	0.3	0.0073	0.8	0.8
CD39 on living cells	0.7	0.7	0.8	1.0
CD73 on living cells	0.3	0.3	0.4	1.0
IgM on living cells	0.6	0.4	0.4	0.8
Living cell mass	1.0	1.0	0.3	0.045
Dead cell mass	0.6	1.0	1.0	1.0
Cell debris mass	0.9	1.0	1.0	0.8
Total cell mass	0.9		1.0	

The metabolites listed are measured in the culture medium. The italicized numbers are p < 0.05.

Colour highlighted numbers are p<0.05. Blue, decreased in ME/CFS compared to HC; Red, increased in ME/CFS compared to HC; FC, fold change; Conc, concentration.

Changes in metabolites (concentrations and fold changes over time) in cultured B cells from HC and ME/CFS patients were measured in cultures with largely B cell growth and also in cultures with largely B cell decay. In **Table 2**, the proliferating and non-proliferating cultures were compared within HC and within the ME/CFS subjects. The clear finding from **Table 2** is the decreased amino acids in the media of proliferating ME/CFS B cells that does not occur in the media of proliferating HC B cells. As there was no increase of total matter created, we surmise that the increased usage of amino acids from the media is for ATP production *via* the mitochondria. The amino acids depleted were essential amino acids, which cannot be created by the cells, so the reduction of essential amino acids may indicate that all amino acids are being used more rapidly in ME/CFS but that the non-essential are being replenished. There is also a significant elevation of lactate in the media of proliferating ME/CFS cells that is not present in HC cells, suggesting the heightened use of glycolysis in ME/CFS. Taken together, it appears that ME/CFS B cells are using media substrates for energy production at a faster rate than HC B cells.

**Table 2:** The significant differences (shown in p-values) between proliferating B cell experiments and non-proliferating B cell experiments from both myalgic encephalomyelitis/chronic fatigue syndrome (ME/CFS) patients and healthy controls are summarized.

	Healthy control		ME/CFS	
	FC	FC of %	FC	FC of %
Acetate	0.1	0.1	0.0049	0.035
Alanine	0.9	0.8	0.2	0.8
Arginine	0.7	0.4	0.1	0.1
Aspartate	0.8	0.6	0.7	0.6
Creatine	0.8	0.8	0.3	0.2
Creatinine	0.8	1.0	0.1	0.1
Formate	0.4	0.2	0.2	0.1
Glucose	0.043	0.1	0.015	0.1
Glutamate	0.9	0.8	0.2	0.3
Glycine	0.8	0.6	0.4	0.7
Histidine	0.046	0.1	0.015	0.2
Isoleucine	0.3	0.3	0.025	0.1
Lactate	0.1	0.2	0.036	0.026
Leucine	0.4	0.4	0.027	0.2
Lysine	0.3	0.4	0.039	0.1
Phenylalanine	0.1	0.2	0.0034	0.021
Proline	0.7	0.6	0.7	0.7
Threonine	0.8	0.8	0.1	0.2
Tryptophan	0.9	0.8	0.0013	0.1
Tyrosine	0.9	0.6	0.013	0.020
Valine	0.2	0.2	0.0019	0.019
Hydroxyproline	0.8	0.4	0.3	0.3
Total metabolite	0.8		1.0	
CD24 on living cells	1.0	0.024	0.4	0.2
CD27 on living cells	0.1	0.010	0.9	0.2
CD38 on living cells	0.7	1.0	0.9	0.8
CD39 on living cells	1.0	0.8	0.9	0.4
CD73 on living cells	0.8	0.2	0.4	0.2
IgM on living cells	0.048	0.6	0.00021	0.4
Living cell mass	0.023	0.6	0.0022	0.039
Dead cell mass	0.030	0.7	0.047	1.3
Cell debris mass	0.0072	0.7	0.2	1.2
Total cell mass	5.05E-10		4.83E-06	

The metabolites listed are measured in the culture medium. The italicized numbers are p < 0.05.

Blue cells, decreased in proliferating B cell experiments compared to non-proliferating; red cells, increased in proliferating B cell experiments compared to non-proliferating; FC, fold change.

## Discussion

Our initial study of B cell immunophenotype in patients with ME/CFS compared to age-matched controls revealed the unexpected finding of a significantly higher frequency (% positive B cells) and expression (MFI) of the GPI-linked surface receptor CD24 on IgD+ B cell sub-populations (16). Although without linkage to the signaling machinery, the widely distributed CD24 molecule has the ability to influence a number of important cellular functions through partnerships with many different signaling molecules, resulting in cell-specific and context-dependent consequences (26). In our previous study, using B cells from healthy individuals, we found that CD24 expression on IgD+ memory but not IgD+ naïve B cells was associated with the phosphorylation of AMPK (20). CD24 appeared to be also closely linked with B cell energy metabolism in that the total CD24 expression had a strong negative correlation with surrogate measures of glycolysis, namely, glucose consumption and lactate production. We have now extended these studies using *in vitro* stimulation of purified B cells from patients with ME/CFS by following changes in B cell immunophenotype, notably CD24 expression and that of the ectonucleotidases CD38, CD39, and CD73. Metabolite fluctuations during culture and changes in mitochondrial mass were also measured in order to explore the aspects of B cell energy metabolism.

Mitochondrial biogenesis is the process whereby mitochondrial mass (MM) is increased due to growth and division and occurs as a result of higher cellular energy demands. It is thus associated with greater glucose uptake and, ultimately, increased ATP production (27, 28). The overall consumption/production of glucose and lactate in cultures from ME/CFS patients and HC was found to be similar over the culture period. Following an initial increase in MM (**Figure 3**), relative MM did not increase or decrease over sequential cycles of proliferation in either ME/CFS and HC B cell cultures. This lack of change in MM indicates that continual usage of aerobic glycolysis was being used more than that of oxidative phosphorylation and electron transport within the mitochondria of B cells from both patient and control sources. This phenomenon has been described as a hallmark for proliferating immune cells, known as the Warburg effect (29–31), where there is a preferred pathway to produce energy rapidly *via* aerobic glycolysis, with increased glucose utilization and lactate production. However, MM within *in vitro*-stimulated B cells from ME/CFS patients was significantly lower compared to age-matched controls throughout multiple cycles, indicative of a decreased utilization of mitochondria for energy production in ME/CFS B cell cultures compared with those from HC. Previous observations in metabolomic studies suggested an aerobic condition but with the minimal utilization of mitochondria for efficient energy production in patients with ME/CFS (11, 32). Proteomic analysis has recently also confirmed a deficiency in ATP production in PBMC from ME/CFS patients, associated with the upregulation of pathways upstream of ATP-synthase (Complex V) which may reflect increased levels of oxidative (increased ROS) stress (33). Our observations using B cell cultures and MitoTracker Red showed less mitochondria in ME/CFS B cells in comparison with those from HC.

We also confirmed the dysregulation of CD24 expression on B cells from ME/CFS patients. Purified B cells from healthy donors exposed to agonists in transwell cultures showed a sharp decrease (~50%) of CD24+ B cells during the first 3 days of culture, but loss of CD24 from cultured B cells from ME/CFS patients was significantly “delayed” following stimulation, with a high proportion of cultured B cells from ME/CFS patients showing a continued expression of CD24 compared with B cells from healthy donors. Loss of CD24 from the naïve B cell surface coincides with B cells entering the cell cycle (16). Despite lacking an intracellular domain, CD24 can access a variety of signaling pathways through its ability to specifically target and regulate the oligomerization of selected cell membrane molecules into lipid rafts (26). In particular, it has been shown that CD24 can alter the localization of the BCR within lipid rafts, thereby affecting intracellular signaling (34, 35). Ligation of the BCR results in a rise in intracellular Ca^2+^ levels leading to the activation of the Ca^2+^/calmodulin-dependent Ser/Thr-specific phosphatase calcineurin and downstream transcription factors, leading to various differentiation pathways (36). Whether intracellular Ca^2+^ levels rose following BCR crosslinking in ME/CFS B cells retaining CD24 or whether the triggering of more downstream signaling pathways was disrupted has not yet been determined. The mechanism of CD24 loss from the cell surface of immature murine B cells and the WEHI-231 cell line in response to antibody-mediated crosslinking of CD24 is mediated *via* the formation and release of CD24+ plasma membrane-derived exosomes (microvesicles) enriched in mitochondrial and metabolism-related products, but whether this occurs following BCR ligation is not known (35).

In our previous study, we showed the dysregulation of CD24 on peripheral blood B cells from ME/CFS patients and that the expression of CD24 on certain (pre- or unswitched) memory subpopulations of human B cells from healthy donors was associated with the phosphorylation of AMPK. The analysis of metabolic profiles using metabolomics has additionally related the metabolic anomalies described in sera from patients with ME/CFS with increased AMPK phosphorylation (37). AMPK phosphorylation is triggered as a response to cell stress to maintain appropriate intracellular ATP generation. It is possible that the use of such catabolic pathways of energy production by B cells in response to stimulation through the BCR, for example, may be necessary to assist/promote cell survival at this critical stage. The links between CD24 expression and different downstream metabolic actions in ME/CFS patients were indeed confirmed by our metabolomic analysis of B cell culture supernatants. We showed, firstly, a clear correlation between CD24 and glucose consumption and lactate production *in vitro* in both HC and ME/CFS patients and, secondly, differences in uptake of metabolites between HC and ME/CFS patients. The increased uptake of amino acids in the ME/CFS cultures during the period of maximum cell growth suggested an increased usage of nitrogen-containing sources for energy production. The particular changes in the metabolites in B cell culture supernatants were remarkably similar to what has previously been published from the metabolomic analysis of serum and urine samples from these patients (5).

Other novel observations involved differences in the relationship between the frequency and expression of CD38+ B cells and the frequency of CD73+ B cells, but not of the other adenosine pathway-linked ectonucleotidase CD39 between cultured ME/CFS and HC B cells. CD38 is widely distributed in the immune system and is often used as an activation marker. It has multiple functions and can act as both a cell adhesion molecule where it is involved in signal transduction and as the enzyme ADP-ribose cyclase which is able to recycle extracellular nucleotides as well as generate cyclic ADP-ribose (cADPR), a potent Ca^2+^-mobilizing second messenger (38, 39). Upon BCR ligation, increased intracellular Ca^2+^ levels are key to controlling effector functions and the fate of B cell immune response (40, 41). We found a significantly increased frequency (%) and expression of CD38 on ME/CFS B cells during the active growth phase of the culture. CD38 expression increases following stimulation in response to the increased need to liberate intracellular Ca^2+^. The reason for a higher percentage (and MFI) of CD38+ B cells in MECFS cultures may possibly reflect a survival advantage, if Ca^2+^ release within ME/CFS B cells is compromised in some way. The liberation of Ca^2+^ through ryanodine receptors from the endoplasmic reticulum is also one of the key regulators of mitochondrial functioning, culminating ultimately in the generation of ATP (42). Some studies have indeed suggested a role for mitochondrial dysfunction or mitochondrial damage as an explanation for changes in energy metabolism in these patients (43). CD38 uses NAD+ for signaling and reduces the cellular availability of NAD+ for important molecules for ATP production.

For cells to survive and grow, an increase of the use of energy substrates to yield a baseline level of ATP is required. These substrates may be glucose, free fatty acids, and amino acids, all of which have been described to be reduced compared to healthy controls in biological fluids from ME/CFS patients (9). The metabolite concentrations in supernatants were measured and used to follow distinct biochemical pathways after the *in vitro* stimulation of B cells. Glucose uptake and lactate production were confirmed as indicators of healthy cell growth during *in vitro* B cell stimulation with no discernible difference in kinetics between patients and controls. The key finding was that increased amino acid consumption occurred in ME/CFS over healthy B cells without a significant difference in B cell quantity or mass. Furthermore, by observing the metabolites consumed by proliferating and non-proliferating B cell cultures from both ME/CFS and HC, we were able to show that the elevated essential amino acid consumption appeared to be necessary for cell growth and the survival of ME/CFS B cells. The use of amino acids as an additional energy source has also been suggested by metabolomic studies of serum from ME/CFS patients and associated with a possible increased degradation of nucleotides to AMP (5). Other authors have proposed that constant depletion of cellular ATP may result in a “Dauer-like” hypometabolic state in these patients (11).

This paper details a deep characterization of B cell behavior *in vitro* comparing ME/CFS patients and healthy controls. The main limitation from this work is the sample size. We present statistically significant findings from this sample size but recommend further validation studies and follow-up work that will utilize this workflow. We are particularly interested in the roles of CD24 and CD38 and the increased substrate usage for B cell proliferation experiments from ME/CFS patients. We unfortunately neglected to identify the morphology and general integrity of the mitochondria, which would have provided more insight into the impact of the mitochondria on substrate consumption from the media. A larger and more comprehensive study of B cells in ME/CFS is recommended in the future.

Many aspects of the dynamic life cycle of human B cells can be readily reproduced *in vitro* using appropriate culture conditions as we have utilized for the studies presented here. From the naïve state following exit from the bone marrow to immunoglobulin production, B cells undergo proliferation, differentiation, and maturation, which involve a complexity of changing energy-producing requirements and mechanisms to limit stress-induced damage. The use of metabolomics to study B cell culture supernatants from ME/CFS patients also revealed shifts in energy homeostasis which paralleled those previously described in their sera. Finally, the anomalies of CD24 and CD38 expression on B cells cultured from ME/CFS patients and their links with metabolic reprogramming and MM may serve as an ideal model for the study of disruption of normal pathways for energy production in patients with ME/CFS.

## Data availability statement

The raw data supporting the conclusions of this article will be made available by the authors without undue reservation.

## Ethics statement

The studies involving humans were approved by the NRES Committee London City Road and Hampstead Research Ethics Committee (REC reference: 14/LO/0388). The studies were conducted in accordance with the local legislation and institutional requirements. The participants provided their written informed consent to participate in this study. Written informed consent was obtained from the individual(s) for the publication of any potentially identifiable images or data included in this article.

## Author contributions

CA and FM designed and conducted experiments and acquired data. CA and PG analyzed metabolomics data. FM analyzed and interpreted immunophenotype data which was then discussed with GC, ML, and VR. SB supplied clinical data and provided all clinical material. GC, CA, and FM wrote the draft manuscript and ML, VR, PG, and SB contributed to the composition/interpretations of results for the completed manuscript. GC was responsible for final layout, and, with CA, the final text of the manuscript. All authors contributed to the article and approved the submitted version.
