# Supplementary figures and images for: In vitro B cell experiments explore the role of CD24, CD38, and energy metabolism in ME/CFS
Source: Front Immunol. 2024 Jan 8;14:1178882. doi: 10.3389/fimmu.2023.1178882 (PMC10800820; doi:10.3389/fimmu.2023.1178882)

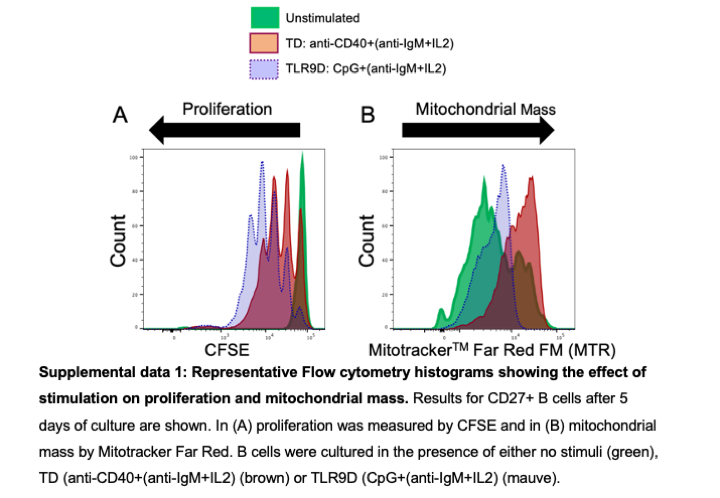

Supplement: Supplementary file 1 [file DataSheet_1.zip › Image 1.TIFF]

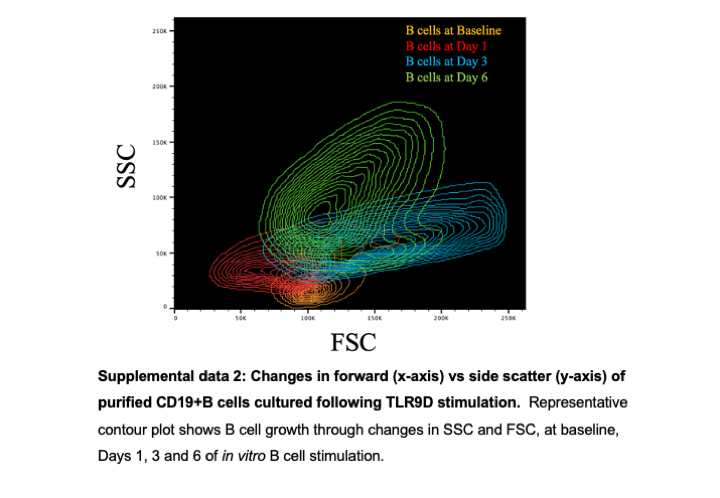

Supplement: Supplementary file 1 [file DataSheet_1.zip › Image 2.TIFF]
